# Insights into the Selectivity Mechanisms of Grapevine NIP Aquaporins

**DOI:** 10.3390/ijms21186697

**Published:** 2020-09-13

**Authors:** Farzana Sabir, Antonella Di Pizio, Maria C. Loureiro-Dias, Angela Casini, Graça Soveral, Catarina Prista

**Affiliations:** 1 Linking Landscape, Environment, Agriculture and Food (LEAF), Departmento de Recursos Biológicos, Ambiente e Território (DRAT), Instituto Superior de Agronomia, Universidade de Lisboa, Tapada da Ajuda, 1349-017 Lisbon, Portugal; mcdias@isa.ulisboa.pt (M.C.L.-D.); cprista@isa.ulisboa.pt (C.P.); 2 Research Institute for Medicines (iMed.ULisboa), Faculty of Pharmacy, Universidade de Lisboa, 1649-003 Lisbon, Portugal; gsoveral@ff.ulisboa.pt; 3 Leibniz-Institute for Food Systems Biology at the Technical University of Munich, Lise-Meitner-Str. 34, 85354 Freising, Germany; a.dipizio.leibniz-lsb@tum.de; 4 Department of Chemistry, Technical University of Munich, Lichtenbergstr. 4, 85748 München, Germany; angela.casini@tum.de

**Keywords:** nodulin 26-like intrinsic proteins, grapevine, ar/R selectivity filter, site-directed mutagenesis, substrate selectivity, homology modeling

## Abstract

Nodulin 26-like intrinsic proteins (NIPs) of the plant aquaporin family majorly facilitate the transport of physiologically relevant solutes. The present study intended to investigate how substrate selectivity in grapevine NIPs is influenced by the aromatic/arginine (ar/R) selectivity filter within the pore and the possible underlying mechanisms. A mutational approach was used to interchange the ar/R residues between grapevine NIPs (VvTnNIP1;1 with VvTnNIP6;1, and VvTnNIP2;1 with VvTnNIP5;1). Their functional characterization by stopped-flow spectroscopy in *Saccharomyces cerevisiae* revealed that mutations in residues of H2/H5 helices in VvTnNIP1;1 and VvTnNIP6;1 caused a general decline in membrane glycerol permeability but did not impart the expected substrate conductivity in the mutants. This result suggests that ar/R filter substitution could alter the NIP channel activity, but it was not sufficient to interchange their substrate preferences. Further, homology modeling analyses evidenced that variations in the pore radius combined with the differences in the channel’s physicochemical properties (hydrophilicity/hydrophobicity) may drive substrate selectivity. Furthermore, yeast growth assays showed that H5 residue substitution alleviated the sensitivity of VvTnNIP2;1 and VvTnNIP5;1 to As, B, and Se, implying importance of H5 sequence for substrate selection. These results contribute to the knowledge of the overall determinants of substrate selectivity in NIPs.

## 1. Introduction

Aquaporins are integral membrane proteins belonging to the major intrinsic protein (MIP) family. They play a crucial role in the transport of water and small solutes through biological membranes and are widely distributed in prokaryotic and eukaryotic organisms [1]. Plant MIPs consist of a large aquaporin family with a unique pattern of subcellular localization [2]. Based on their localization and phylogenetic analysis, they are grouped in five major subfamilies: plasma membrane intrinsic proteins (PIPs), tonoplast intrinsic proteins (TIPs), nodulin 26-like intrinsic proteins (NIPs), small, basic intrinsic proteins (SIPs), and X (uncharacterized) intrinsic proteins (XIPs) [2]. Two additional minor subfamilies, HIPs (hybrid intrinsic proteins) and GlpF-like intrinsic proteins (GIPs), have also been identified in primitive plant species like mosses, lycopods, and some green algae, which are considered to be lost during evolution [3]. Loss of XIPs in monocots, and NIPs in green algae, have also been reported [3,4]. The existence of a large number of aquaporins in plants has been explained by various processes of gene duplication during evolution, resulting in the functional diversity of duplicated plant aquaporins [5].

The multiple roles of plant aquaporins have been associated with their interaction with solutes at the molecular level. The advancements in membrane protein structural biology have allowed the determination of the three-dimensional atomic structures of aquaporins from different organisms, including plants, revealing their characteristic hourglass structure in the membrane [6,7]. The structural architecture of aquaporins are conserved from archaea to humans [8]. They exist as tetramers in the membrane, each monomer consisting of six transmembrane helices (H1 to H6) and five intracellular and extracellular loops (A to E). The pore is characterized by two constriction regions. Two NPA (asparagine, proline, and alanine) motifs form the first constrictions at the center of the pore. The second constriction is composed of four residues from H2, H5, and loop E (LE1 + LE2), and referred as aromatic-arginine (ar/R) region due to the presence of a conserved arginine residue in loop E and the frequent occurrence of aromatic residues at H2 [9]. The ar/R constriction is located at the periplasmic side of the pore, 8–9 Å away from the first NPA motif [10]. The ar/R region represents the narrowest constriction of the pore and a major checkpoint for solute permeability [8], acting as a selectivity filter for substrates based on steric effects. In plants, the amino acid composition of ar/R filter is the least conserved with respect that of their mammalian and microbial counterparts [11,12], pointing towards its more diverse substrate profile and novel physiological relevance.

Among all plant aquaporins, NIPs exhibit the greatest degree of divergence in the substrate profile like glycerol, boron, arsenite, ammonia, silicon (reviewed by Reference [13]), and aluminum [14] with lower intrinsic water conductivity. The diverse substrate selectivity of NIPs has been associated with their unique composition of amino acid residues at the ar/R selectivity filter. Based on these residues, NIPs have been categorized into three functional groups (NIP-I, NIP-II, and NIP-III), which have a distinct pore size. The ar/R region of NIP-I proteins is characterized by W-V/I-A-R forming more hydrophilic and smaller pore (diameter ~2.8 Å) allowing the conductance of water, glycerol, and lactic acid (reviewed by Reference [13]). The selectivity filter of NIP-II proteins consists of (T/A-A/I/V-G/A-R), resulting in larger pore (diameter ~3.4 Å) with relatively more hydrophobic residues, permeable to larger solutes like glycerol and boric acid, but not permeable to water [15]. The amino acid composition of NIP-III ar/R region (G-S-G-R) provides the widest pore (≥6 Å), permeable to solutes with larger diameter, like silicic acid (4.38 Å), but not permeable to the relatively smaller glycerol molecule [16]. Similarly, our recent studies also demonstrated the above mentioned substrate profile of *Vitis vinifera* L. NIPs belonging to these three groups, NIP-I (VvTnNIP1;1) NIP-II (VvTnNIP6;1) [17] and NIP-III (VvTnNIP2;1) [18]. Their functional characterization in *aqy-null Saccharomyces cerevisiae* demonstrated that VvTnNIP1;1 is an aquaglyceroporin, whereas VvTnNIP6;1 has lower glycerol conductance than VvTnNIP1;1. On the other hand, VvTnNIP2;1 and VvTnNIP5;1 did not transport water and glycerol, yet VvTnNIP2;1 was demonstrated as Si channel. These results imply that functionally, as well as structurally, distinct NIP groups may have different physiological significance in plants in terms of membrane solute transport and water balance.

The critical role of the ar/R region in many plant MIPs subfamilies, like PIPs [19], TIPs [20], and XIPs [21], has been elucidated by the substitution of key amino acids (reviewed by Reference [22]). However, only a few studies have addressed NIPs, despite being considered as crucial channels for transport of various beneficial (like B and Si), as well as toxic (like As), metalloids in plants [23,24,25,26]. Thus, the present study aimed to explore the role of ar/R filter in the regulation of substrate selectivity in grapevine NIPs [17,18]. Site directed mutagenesis in their r/R region was performed and the resulting functional divergence caused by possible changes in the pore structural architecture was investigated by stopped-flow fluorescence [27]. Furthermore, the experimental results were complemented with structural bioinformatics investigations aiming to rationalize the effect of residue variations on protein structure. Additionally, the influence of ar/R filter substitution on putative conductance of metalloids was investigated by yeast sensitivity/tolerance growth assays. The obtained findings will further broaden the molecular and physiological understanding of functions of different NIP isoforms, as well as plant aquaporins, in general.

## 2. Results and Discussion

To investigate the functional significance of the two conserved residues in putative ar/R filter (H2 and H5) of the pore region of NIP subgroups (I, II, and III) proteins, amino acid substitution within the NIP subgroup was performed by site-directed mutagenesis in VvTnNIP1;1, VvTnNIP2;1, VvTnNIP5;1, and VvTnNIP6;1 [17]. In the case of VvTnNIP6;1, a mutated version (VvTnNIP6;1M) with extended C-terminal, possessing water conductivity and improved glycerol permeability, was chosen, to investigate the influence of ar/R substitution on both glycerol and water transport. To avoid any confusion, VvTnNIP6;1M will be further mentioned as VvTnNIP6;1.

**Figure 1:**
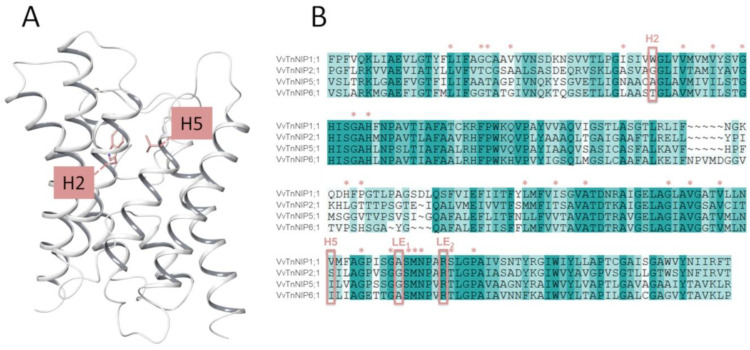
(**A**) Representation of the grapevine VvTnNIP1;1 structural model with H2 and H5 residues as pink sticks. (**B**) Sequence alignment of grapevine VvTnNIP1;1 (residues 43–259), VvTnNIP2;1 (residues 68–259), VvTnNIP5;1 (residues 68–283), and VvTnNIP6;1 (residues 75–292). The aromatic/arginine (ar/R) selectivity filter is framed, and additional conserved channel residues are annotated with stars (*). NIP = Nodulin 26-like intrinsic proteins.

In the present study, the H2 and H5 residues of the ar/R selectivity filter of grapevine NIPs were considered for site-directed mutagenesis study, while LE1 and LE2 residues were maintained because they are highly conserved in NIPs. Thus, the amino acid residues at H2/H5 of VvTnNIP1;1 (NIP-I group) were interchanged with VvTnNIP6;1 (NIP-II group), whereas the amino acid residues of VvTnNIP2;1 (NIP-III group) were interchanged with VvTnNIP5;1(NIP-II group). At H2, W was substituted with T in the case of VvTnNIP1;1; G with A in VvTnNIP2;1, and at H5, V was substituted with I and S with I, respectively. Figure 1 shows H2 and H5 positions mapped on the VvTnNIP1;1 structure, and the sequence alignment of analyzed NIPs. Overall, these NIPs share a sequence identity ranging from 43% (between VvTnNIP2;1 and VvTnNIP6;1) to 66% (between VvTnNIP5;1 and VvTnNIP6;1), respectively.

### 2.1. Water and Glycerol Transport Activity of Native VvTnNIP1;1 and VvTnNIP6;1, and Their ar/R Filter Mutant Variants

**Figure 2:**
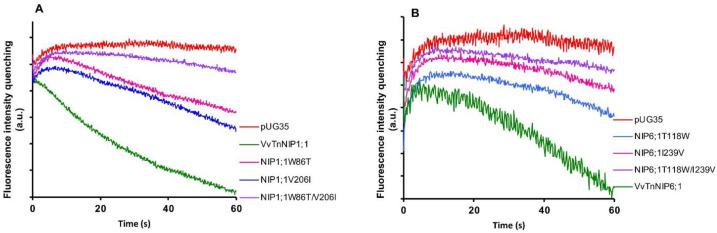
Glycerol permeability of yeast cells transformed with grapevine NIPs and their mutants in H2, H5, H2/H5 helices. Fluorescence quenching after a hyperosmotic shock applied to yeast cells expressing (**A**) VvTnNIP1;1 and their single mutants W86T, V206I, and double mutant (W86T/V206I). (**B**) VvTnNIP6;1 and their single mutants T118W, I239V, and double mutant (T118W/I239V). Yeast strain with an empty vector (pUG35) was considered as the negative control.

Stopped-flow fluorescence spectroscopy was employed to compare the water and glycerol transport activity of yeast cells expressing various ar/R mutant constructs of functional grapevine NIPs, namely VvTnNIP1;1 and VvTnNIP6;1 from the previous study [17]. In detail, we constructed three mutated versions of VvTnNIP1;1, either at H2 (W86T) or H5 (V206I) positions, with a double mutant at both H2 and H5 positions (W86T/V206I) in the present study. Similarly, three mutants of VvTnNIP6;1 were generated at H2 (T118W), H5 (I239V), and H2/H5 (T118W/I239V) positions. The obtained fluorescence signals corresponding to cell volume changes induced by glycerol movements through native grapevine functional NIPs (VvTnNIP1;1 and VvTnNIP6;1), and their constructed mutants are shown in Figure 2. According to the obtained results, as expected the various mutations did not affect the trafficking of grapevine NIP-GFP homologs in the yeast membrane (Figure S1) but did affect their permeability to glycerol.

**Figure 3:**
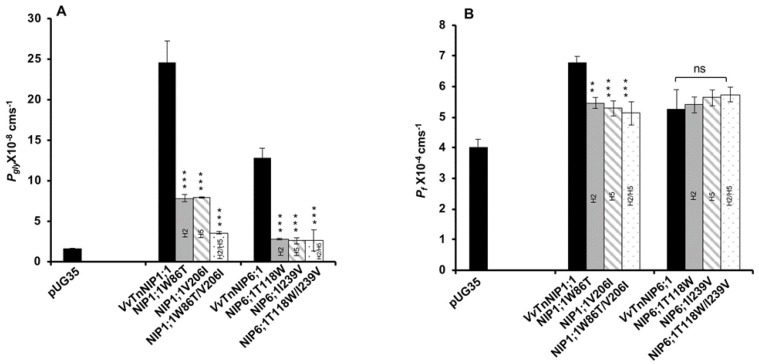
Effect of point mutation in the ar/R selectivity filter of grapevine VvTnNIP1;1 and VvTnNIP6;1 on (**A**) glycerol (*P_gly_*), (**B**) water (*P_f_*), and permeability coefficients. The permeabilities were measured in yeast strains expressing functional grapevine NIPs, and their mutants in H2, H5, and H2/H5 helices. Cells expressing empty vector pUG35 were considered as a negative control. Data represent the mean ± SD of three independent experiments with ten technical replicates. Statically significance differences are shown as an asterisk, calculated by *t*-test (** *p* < 0.01 and *** *p* < 0.001); no significant differences are shown as ns.

Glycerol transport activity of VvTnNIP1;1 (*P_gly_*: 24.5 ± 2.73 × 10^−8^ cm s^−1^, *E_a_*: 6.93 ± 0.22 kcal mol^−1^) and VvTnNIP6;1 (*P_gly_*: 12.8 ± 1.2 × 10^−8^ cm s^−1^, *E_a_*: 8.60 ± 0.50 kcal mol^−1^) were significantly reduced in all mutant strains, the lower permeabilities (*P_gly_*) being consistent with the higher activation energies (*E_a_*) (Figure 3A) (Table 1). The reduction in glycerol permeabilities in VvTnNIP1;1 (W86T: 7.84 ± 0.45 × 10^−8^ cm s^−1^, V206I: 7.9 ± 0.08 × 10^−8^ cm s^−1^) and VvTnNIP6;1 (T118W: 2.8 ± 0.09 × 10^−8^ cm s^−1^, I239V: 2.57 ± 0.38 × 10^−8^ cm s^−1^) was observed up to ~68% and ~79% due to point mutation at H2 and H5 residues, respectively, whereas double mutation in H2 and H5 residues of VvTnNIP1;1 (W86T/V206I: 3.58 ± 0.17 × 10^−8^ cm s^−1^) and VvTnNIP6;1 (T118/I239V: 2.63 ± 1.3 × 10^−8^ cm s^−1^) sharply reduced the permeability up to ~85% (Figure 3A). In contrast, water permeability of VvTnNIP1;1 (6.78 ± 0.21 × 10^−4^ cm s^−1^) was slightly reduced in all of its mutant homologs (W86T: 5.47 ± 0.18 × 10^−4^ cm s^−1^, V206I: 5.29 ± 0.25 × 10^−4^ cm s^−1^, and W86T/V206I: 5.12 ± 0.38 × 10^−4^ cm s^−1^) with consistent higher activation energies (*E_a_*) for water transport (Figure 3B) of mutant homologs compared with their native proteins (Table 1), whereas water permeability was unaffected in VvTnNIP6;1 (5.25 ± 0.65 × 10^−4^ cm s^−1^) mutants (T118W: 5.4 ± 0.26 × 10^−4^ cm s^−1^, I239V: 5.23 ± 0.26 × 10^−4^ cm s^−1^, and T118W/I239V: 5.74 ± 0.24 × 10^−4^ cm s^−1^) expressing strains (Figure 3B).

We recently demonstrated that VvTnNIP1;1 is an aquaglyceroporin with high glycerol and moderate water permeability, respectively. Differently from VvTnNIP1;1, VvTnNIP6;1 is an aquaglyceroporin with low water transport capability, although featuring less pronounced glycerol permeation than VvTnNIP1;1 [17]. The ar/R filter of aquaglyceroporin VvTnNIP1;1 is comprised of WVAR. This unique combination in NIP-I group is a hybrid sequence of residues between aquaporins and aquaglyceroporins, where valine and alanine residues pair along with conserved tryptophan and arginine and possibly configure an amphipathic surface to interact with the hydrocarbon backbone of glycerol, as well as with its hydroxyl groups [23]. The H2 tryptophan residue of VvTnNIP1;1 is also conserved in aquaglyceroporin *E. coli* glycerol facilitator (GlpF), and its substitution to threonine (W86T) resulted in reduced permeability, while water permeability was only slightly affected (Figure 3B). Similar to our finding, glycerol conductance in the aquaglyceroporin LIMP2 (NIP-I group) of *Lotus japonicus* was largely lost due to tryptophan substitution in the H2 filter, while water permeability was only slightly reduced [23]. These results suggest the importance of the H2 residue, specifically for glycerol transport in the NIP-I group. Furthermore, the substitution of valine to isoleucine (V206I) in helix 5 of VvTnNIP1;1 also significantly reduced the glycerol permeability. So far, the significance of the H5 residue for glycerol transport by aquaglyceroporins of NIP-I group has not been elucidated. Our results indicate that not only the residues at H2 but also those at H5 is crucial for glycerol transport in the grapevine NIP-I group.

Likewise, the amino acid substitution in VvTnNIP6;1 at H2/H5 position (T118W and I239V) also resulted in declining the glycerol transport without affecting the water permeability. This finding is opposite to what was observed in the case of a previously reported AtNIP6;1 ar/R filter (AIAR) mutation study [26], in which substitution of alanine (at H2 position of NIP-II) to tryptophan of Nod26 (NIP-I) resulted in enhanced water permeability, while glycerol permeability was mostly unaffected. It is noteworthy that VvTnNIP6;1 has threonine instead of alanine at H2 position, and its substitution by tryptophan of VvTnNIP1;1 significantly reduced glycerol conductance. This substitution of NIP-II group residue by NIP-I residues did not acquire the higher water permeability equivalent to NIP-I group. This observation is similar to the previously reported results by exchanging the ar/R residue of an aquaglyceroporin LIMP2 with a water selective aquaporin LIMP1. This substitution could not enhance the water permeability, despite the much wider predicted pore average diameter (5 Å), which can accommodate more than one water molecule [23,26]. Moreover, members of NIP-III group, including grapevine VvTnNIP2;1, assumed to have the largest pore aperture and able to transport bulkier substrates like silicic acid, also have restricted selectivity for relatively smaller water and glycerol molecules [16,18].

### 2.2. Analysis of Homology Models of Native VvTnNIP1;1 and VvTnNIP6;1, and Their ar/R Residue Mutants

**Figure 4:**
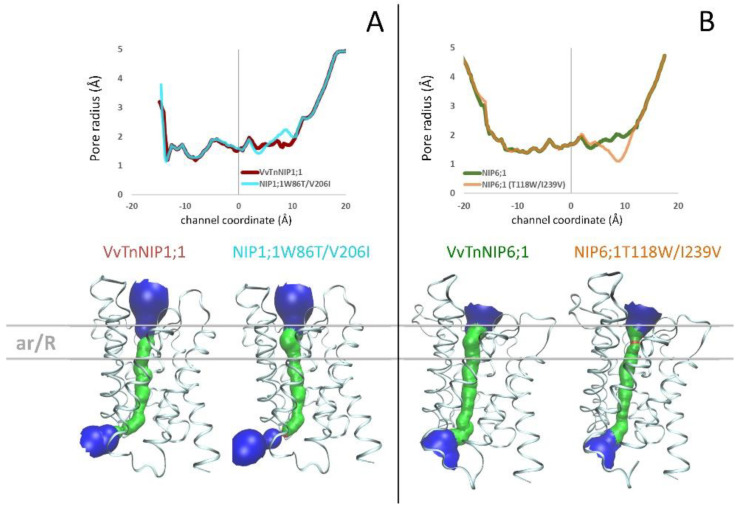
(**A**) Pore radius profiles of native VvTnNIP1;1 (red line) and of double mutant NIP1;1W86T/V206I (cyan line) are shown in the upper panel; 3D structures and pore surfaces of VvTnNIP1;1 and of NIP1;1W86T/V206I in the bottom panel. (**B**) Pore radius profiles of native VvTnNIP6;1 (green line) and of double mutant NIP6;1T118/I239V (orange line) are shown in the upper panel; 3D structures and pore surfaces of VvTnNIP6;1 and of NIP6;1T118W/I239V in the bottom panel.

The structure of *Arabidopsis thaliana* AtTIP2;1 [28] was selected as the template structure of the homology modeling. Comparing the resulting VvTnNIP1;1 and VvTnNIP6;1 models with their respective mutants NIP1;1W86T/V206I and NIP6;1T118W/I239V, we did not observe significant changes of the channel radius profiles (Figure 4). Specifically, we observed a slight increase (ca. +0.4 Å) of the channel radius of NIP1;1T118W/V206I compared to that of VvTnNIP1;1 (Figure 4A) and a slight reduction (ca. −0.8 Å) of the channel radius of NIP6;1T118W/I239V compared to VvTnNIP6;1 (Figure 4B). The pore restriction observed for NIP6;1T118W/I239V could explain the reduced glycerol permeability compared to VvTnNIP6;1. However, mutations in VvTnNIP1;1 led to decreased glycerol permeability instead of an expected increase due to larger pore aperture. Similarly, NIP6;1T118W/I239V mutant did not acquire water permeability despite carrying the selectivity filter of the water channel VvTnNIP1;1.

**Figure 5:**
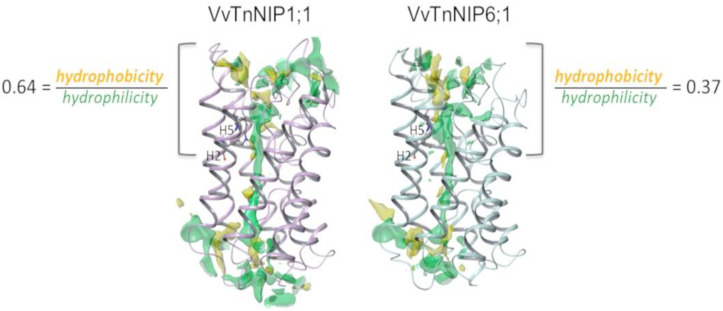
Hydrophobic (**yellow**) and hydrophilic (**green**) regions of the channel in VvTnNIP1;1 and VvTnNIP6;1.

Therefore, structural features determining substrate selectivity might not be restricted to the ar/R filter. Indeed, the distribution of hydrophobic and hydrophilic regions differs in the channel of VvTnNIP1;1 model compared to that of VvTnNIP6;1 (Figure 5). In general, the overall ratio of hydrophobicity/hydrophilicity is quite similar, i.e., 0.47 and 0.41 for VvTnNIP1;1 and VvTnNIP6;1, respectively. However, the calculated ratio on the upper region of the pore indicates increased hydrophobicity (ratio of 0.64) for the aquaglyceroporin VvTnNIP1;1 compared to VnTnNIP6;1 (ratio of 0.37), implying that the higher hydrophobicity may be a possible reason for the higher glycerol permeability through VvTnNIP1;1 (Figure 3A). Nevertheless, exchanging the H2 and H5 residues of the two NIPs, the ratios are not reversed: we observed a decrease of the hydrophobicity/hydrophilicity ratio for NIP1;1W86T/V206I to 0.50 and an increase of the ratio NIP6;1T118W/I239V to 0.53, in line with the observed similar range of glycerol permeability in double mutants NIP1;1W86T/V206I (3.58 ± 0.17 × 10^−8^ cm s^−1^) and NIP6;1T118W/I239V (2.63 ± 1.3 × 10^−8^ cm s^−1^). This suggests that the diverse composition of the NIP pore residues (Figure 1B) may influence the physicochemical properties of the channel.

**Figure 6:**
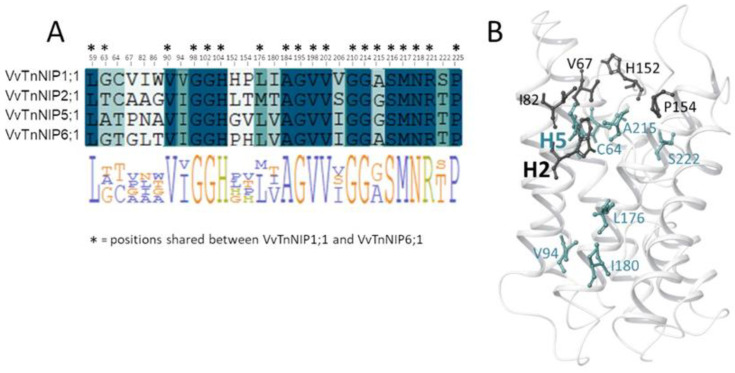
(**A**) Sequence alignment annotated with VvTnNIP1;1 numbering of the 29 channel residues colored from white to blue according to the degree of residue similarity (positions shared between VvTnNIP1;1 and VvTnNIP6;1 are annotated with stars). (**B**) Diverse NIP channel residues (similar and dissimilar residues are colored in green and in black, respectively) mapped on the 3D structure of VvTnNIP1;1.

Analysis of the pore residues as annotated in Figure 1B showed that 19 residues are shared between VvTnNIP1;1 and VvTnNIP6;1 (annotated in the sequence alignment in Figure 6A), and 16 are conserved in all NIPs selected for this study (highlighted in blue in the sequence alignment in Figure 6A). Instead, 8 of the 13 diverse residues (positions 63, 64, 94, 176, 180, 206, 215, 222, according to VvTnNIP1;1 numbering, highlighted in green in the sequence alignment in Figure 6A) share a certain chemical similarity, whereas 5 residues (at positions 67, 82, 86, 152, 154, in white in Figure 6A) are dissimilar. Mapping the 13 diverse residues on the VvTnNIP1;1 structure model, the most dissimilar residues (represented as black sticks in Figure 6B) cluster in the upper region of the ar/R filter.

This analysis suggests that residues at the entrance of the channel may contribute to the different hydrophobicity/hydrophilicity and surface charge distribution—as observed for VvTnNIP1;1 and VvTnNIP6;1—and, together with residues of the ar/R filter, may play a relevant role in determining NIP substrate selectivity. It is worth mentioning that other computational studies on the interaction of human aquaglyceorporins with glycerol and selective small-molecule inhibitors highlighted the pivotal role of the chemical composition of the extracellular AQP’s pocket in modulating the affinity of the protein channel towards its substrate/inhibitor via the establishment of numerous non-covalent interactions [29,30]. The latter cannot be excluded to play a role in the selectivity of NIPs for certain substrates.

### 2.3. Influence of ar/R Residue Mutation on Putative Transport of Other Substrates in Grapevine NIPs

Our recent study revealed that native grapevine NIPs might have a putative role in transport of arsenic, boron and selenium [17]. Yeast cells expressing VvTnNIP1;1 and VvTnNIP6;1 showed sensitivity towards arsenous and boronic acid. These two strains were also sensitive to selenium, in the form of selenite, where VvTnNIP1;1 expressing cells were more sensitive than VvTnNIP6;1 expressing cells. On the other hand, VvTnNIP5;1 showed less or no sensitivity to all the mentioned substrates [17]. Additionally, a recent study unveiled VvTnNIP2;1 as a silicon channel [18]. Based on these results, phenotypic growth assays of yeast strains expressing ar/R mutants were performed to investigate the influence of ar/R amino acid substitution on the conductance of other small solutes like arsenic, boron, and selenium species.

**Figure 7:**
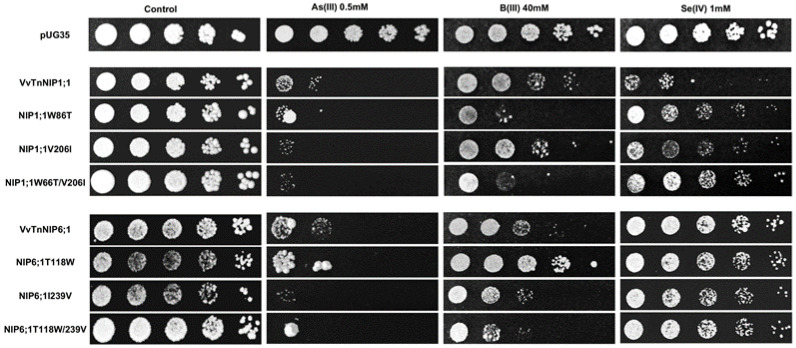
*S. cerevisiae aqy-null* strain expressing grapevine native NIPs (VvTnNIP1;1 and VvTnNIP6;1), and their ar/R filter mutants. Native strains exhibited sensitivity toward metalloids (As, B, and Se), which was altered by amino acid substitution at H2/H5 residue. Yeast strain transformed with empty plasmid pUG35 plasmid was considered as control. Cells were spotted in 10-fold dilution on plates containing the indicated concentration of test substrates. Minimal media plate without any additional substrate is considered as control. Growth was recorded after one week at 28 °C. Photographs are representative of at least two independent experiments having two replicate plates showing consistent results.

**Figure 8:**
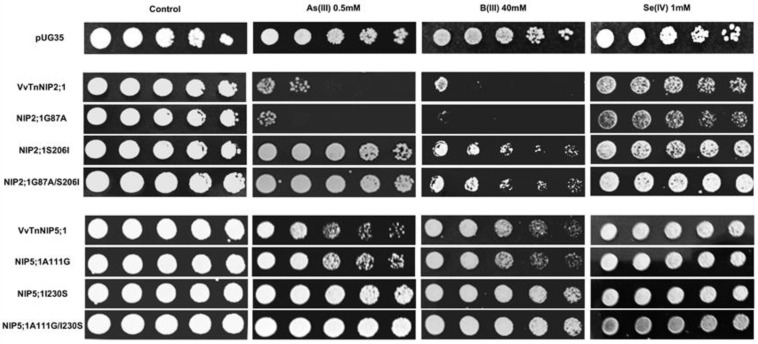
*S. cerevisiae aqy-null* strain expressing grapevine native NIPs (VvTnNIP2;1 and VvTnNIP5;1), and their ar/R filter mutants. Native strains exhibited sensitivity toward metalloids (As, B, and Se), which was altered by amino acid substitution at H2/H5 residue. Yeast strain transformed with empty plasmid pUG35 plasmid was considered as control. Cells were spotted in 10-fold dilution on plates containing the indicated concentration of test substrates. Minimal media plate without any additional substrate is considered as control. Growth was recorded after one week at 28 °C. Photographs are representative of at least two independent experiments having two replicate plates showing consistent results.

The results obtained by interchanging the ar/R residues between VvTnNIP1;1 and VvTnNIP6;1 homolog showed that their sensitivity toward arsenous acid was not greatly affected (Figure 7). Yeast cells expressing the various ar/R mutants of VvTnNIP1;1 (W86T, V206I, and W86T/V206I) and VvTnNIP6;1 (T118W, I239V, and T118W/I239V) grew at a slightly lower rate than yeast cells transformed with their native homologs in the presence of AsOH_3_. On the other hand, in the presence of BOH_3_, altered sensitivity was observed in H2 residue-mutants of NIP1;1W86T and NIP6;1T118W. The sensitivity was affected in both mutants in opposite ways: while the expression of NIP1;1W86T caused higher sensitivity, conversely, NIP6;1T118W expression resulted in lower sensitivity in yeast (Figure 7). In the case of selenium, mutations at H2 and H5 positions both caused a loss of sensitivity in VvTnNIP1;1 strain, which was strongly detected in the double mutated strain (W86T/V206I). The other less or not sensitive strains (VvTnNIP6;1 and VvTnNIP5;1, respectively) for selenium [17] were not affected by substitution at the ar/R residue (Figure 7 and Figure 8).

In the present work, phenotypic growth of VvTnNIP2;1 was affected by arsenous and boronic acids (Figure 8). Our recent study also indicated the role of these isoforms in the transport of arsenic and boron, along with silicon in grapevine [18]. Moreover, a slight sensitivity of VvTnNIP2;1 expressing cells was also observed in the presence of selenium (Figure 8). Our results herein indicate that substitution at H2 position (G87A) did not affect the sensitivity toward these substrates. Instead, mutations at the H5 position, when serine, a small polar amino acid with H-bonding ability, was substituted by isoleucine, bulkier and non-polar (S206I), the sensitivity of expressing cells was entirely lost towards all the three substrates (Figure 8). A similar pattern in the loss of sensitivity was observed due to mutation at H5 position in VvTnNIP5;1, albeit to a lower extent (Figure 8). Our finding clearly suggests the significance of H5 residue in the transport of As, B, and Se through VvTnNIP2;1 (NIP-III group) and VvTnNIP5;1 (NIP-II group), whereas H2 residues did not seem to play a role in the selection of these substrates.

Our results are in accordance with a previous study, in which substitutions of H5 residues in OsLsi (OsNIP2;1, NIP-III group) and AtNIP5;1 (NIP-II) were shown crucial for the selection of metalloids in these NIP groups [24]. However, the ar/R residues seemed crucial only for boron, silicon, and germanium selection, and not for arsenic in OsLsi [24], and in HvNIP2;1 [25]. Our result indicates that ar/R filter (H5 residue) is also critical for VvTnNIP2;1 mediated arsenic transport in grapevine. As shown in Figure 8, the sensitivity of NIP2;1 was entirely lost towards arsenous acid due to the mutation at the H5 position (S206I). In addition, the strain expressing double mutations at H2 and H5 positions (G87A/S206I) lost sensitivity to As. A similar pattern in the loss of sensitivity was observed in VvTnNIP5;1 due to mutation at H5 position, although, to a lower extent.

Interestingly, a western blot analysis showed a different pattern of native and H5-mutated OsLsi protein, suggesting that H5 substitution affects the post-translational modification. However, this effect was not evident in AtNIP5;1 of the NIP-II group, suggesting that regulation of substrate selection involves distinct factors and mechanisms in different NIP groups. Nevertheless, VvTnNIP6;1 (TIAR), which also belongs to NIP-II group, showed different substrates profile compared to VvTnNIP5;1, and it was affected by both H2 and H5 residue substitution (Figure 8), which leaves the open question on group-specific regulatory mechanisms for substrate selection. In general, the results indicate that the effect on substrate selection due to ar/R substitution is not group-specific, although it is unique to each NIP and substrate.

## 3. Materials and Methods

### 3.1. Construction of Grapevine NIP Mutants and Their Transformation in S. cerevisiae

Amino acid residues at H2/H5 of VvTnNIP1;1 with VvTnNIP6;1, as well as VvTnNIP2;1 with VvTnNIP5;1, were interchanged (Table 2). Mutations in putative ar/R filters of NIPs were introduced by whole plasmid PCR using complementary primers listed in Table S1. Previously constructed pUG35 plasmid encoding C-terminal GFP-tagged grapevine NIPs (VvTnNIP1;1, VvTnNIP2;1, VvTnNIP5;1 and VvTnNIP6;1) were utilized as template [17]. In each NIP, single mutants at H2/H5 positions (VvTnNIP1;1-W86T and -V206I, VvTnNIP2;1-G87A and -S206I, VvTnNIP5;1-A111G and -I230S and VvTnNIP6;1-T118W and -I239V), and double mutants combining the mutations at H2 and H5 positions (VvTnNIP1;1-W86T/V206I, VvTnNIP2;1-G87A/S206I, VvTnNIP5;1-A111G/I230S, VvTnNIP6;1-T118W/I239V) were constructed. The PCR products were digested with DpnI enzyme prior to transformation into E. coli to digest the methylated parental plasmid DNA. Mutations in transformants were verified by plasmid DNA sequencing. The selected plasmids were transformed into the S. cerevisiae aqy-null strain [31], and the GFP-tagged aquaporins were localized as described previously [32].

### 3.2. Functional Assays of Grapevine NIPs, and Their ar/R Mutants in S. cerevisiae

Water and glycerol permeabilities of native VvTnNIP1;1 and VvTnNIP6;1, and their constructed mutants were measured by stopped-flow fluorescence spectroscopy (HI-TECH Scientific PQ/SF-53), as previously described [27,32,33]. Briefly, osmotic water permeability (*P_f_*) was measured by subjecting the yeast cells equilibrated in 1.4 M sorbitol buffer and loaded with the fluorophore precursor 5-(and-6)-carboxyfluorescein diacetate (CFDA), to a hyperosmotic shock with 2.1 M sorbitol. The inwardly generated sorbitol gradient leads to water efflux and cell shrinkage, inducing quenching of the fluorescent dye with consequent changes of the signal output. The rate constant (k) was calculated by fitting the obtained signals to a single exponential. The osmotic water permeability coefficient (*P_f_*) was estimated by *P_f_* = *k (V_o_/A)(1/V_w_(osm_out_)_∞_)*, where *V_o_/A* is the initial cell volume to area ratio, *V_w_* is the molar volume of water, and *(osm_out_)_∞_* is the final medium osmolarity after the osmotic shock.

Glycerol permeability (P_gly_) was measured by subjecting the prepared yeast cells to a hyperosmotic shock with 2.1 M glycerol. The hyperosmotic shock leads to water efflux causing rapid cell shrinkage. Further, glycerol and consequent water influx cause cell re-swelling. The re-swelling rate by glycerol influx was estimated as the slope of a linear regression fit. The glycerol permeability coefficient (P_gly_) was calculated from P_gly_ = m(V_o_/A), where m is the linear slope fitted to the signal of glycerol influx. Osmotic shocks were subjected at 23 °C for both water and glycerol permeabilities. Activation energies (E_a_) were evaluated from the slope of Arrhenius plots (lnP_f_ or lnP_gly_ as a function of 1/T). For that, cells were subjected to osmotic shocks at various temperatures, ranging from 10 °C to 35 °C.

### 3.3. Homology Modeling

Homology models of VvTnNIP1;1, VvTnNIP6;1 and their double mutants were built using the *Arabidopsis thaliana* AtTIP2;1 (PDB ID: 5I32) structure as a template [28]. The template structure shares a sequence identity of 30%, and 32% with VvTnNIP1;1 (residues 43–259) and VvTnNIP6;1 (residues 75–292), respectively. Sequence alignment and homology modeling were performed with Prime (Schrödinger Suite 2019_4, Prime, Schrödinger, LLC, New York, NY, 2019). 3D models with water molecules inside the pore channel in the template structure were then optimized with the Protein Preparation Wizard tool from Schrödinger at a pH range of 6–8 and minimized to a derivative convergence of 0.05 kJ/mol-Å using the Polak-Ribiere Conjugate Gradient (PRCG) minimization algorithm, the OPLS2005 force field and the GB/SA water solvation model implemented in MacroModel (Schrödinger Suite 2019_4, MacroModel, Schrödinger, LLC, New York, NY, 2019). In order to construct the mutant models, we manually substituted the coordinates of the H2 and H5 positions of ar/R filter of the VvTnNIP6;1 model with those of the VvTnNIP1;1 model (NIP6;1T118W, NIP6;1I239V and NIP6;1T118W/I239V), and vice versa (NIP1;1W86T, NIP1;1V206I and NIP1;1 W86T/V206T).

SiteMap (Schrödinger Suite 2019_4, SiteMap, Schrödinger, LLC, New York, NY, 2019), with fine grid setting, was used to calculate the hydrophobic and hydrophilic regions in the NIP channels. HOLE was used to calculate the radius profiles and visualize the channel of VvTnNIP1;1 and VvTnNIP6;1 and the double mutants.

### 3.4. Growth Analysis of Yeast Strains Expressing Mutant NIPs in the Presence of Atypical Substrates

Drop tests were performed for yeast tolerance/sensitivity growth assessment, as described previously [17]. In brief, actively growing yeast cells were harvested at the early exponential phase (OD_640 nm_ ≈ 0.6–0.8). After centrifugation, the cells were re-suspended in sterile distilled water at OD_640 nm_ ≈ 1.0, and 10-fold serially diluted. Using a replica platter, 3 µL cell suspension was spotted on plates containing YNB solid medium with test substrates, arsenic (As^III^) as arsenous acid (As(OH)_3_), boron (B^III^) as boric acid (B(OH)_3_), and selenium (Se^IV^) as selenite (HSeO_3_^−^), which were previously shown to be putatively transported by the chosen NIPs [17]. Yeast strain with an empty vector (pUG35) was considered as the negative control. Differences in growth were scored after one week of incubation at 28 °C.

## 4. Conclusions

On the basis of the obtained results, it could be concluded that amino acid residues in ar/R selectivity filter of grapevine NIPs are critical determinants of the transport rate of glycerol, as well as of selectivity profiles, for metalloids, like arsenic, boron, and selenium. Subtle changes, like single point mutations at H2/H5, were sufficient to reduce or abolish the conductance of substrates other than water, as described in a previous study [26], suggesting that mutations in this region may affect the overall conformation of the channel. However, interchanging the amino acid residue at the ar/R filter between two grapevine NIPs was not sufficient to exchange their substrate conductivity.

The combined use of homology modeling and functional analysis indicated that distinct residues at the entrance of the channel influence the hydrophobicity/hydrophilicity of the pore surface. Certainly, other factors, like the orientation of the key residues, physicochemical properties, and proper folding of the channel, may play an important role in tuning the substrate selection, in addition to the ar/R filter composition. It should also be noted that aquaporin homologs of smaller organisms, with entirely different ar/R residues, efficiently transport water, glycerol, and other metalloids [34,35,36] (also see review in Reference [37]). Moreover, previous studies on human AQPs showed that variations in the polarity of certain areas at the entrance of the channels may have an important effect in facilitating the permeation of a specific substrate [38] or the approach of a selective inhibitors to the pore [39].

Recent metadynamics studies showed that glycerol permeation in human aquaporins experiences the highest energy barrier in the NPA filter. Overall, the substrate transport was found to critically depend on bond switches within a dynamic hydrogen-bond scaffold created by the interplay of glycerol, water molecules and pore amino acid residues, disclosing a novel scenario, in which substrate molecules exploit an existing water conduction mechanism [30]. Therefore, it would be worth to carefully evaluate also the effects of the NPA selectivity filter on grapevine NIPs’ substrate selectivity.

It should also be noted that point mutations can also results in a more dramatic conformational change than what is expected and observed in classical homology modeling studies. For example, in AtPIP2;1, the substitution of ar/R residue (F87H) at H2 position, resulted in a blocked pore because it twisted into the pore lumen, caused by the neighboring T55 side chain, which is not even involved in pore configuration but exist in juxtaposition to pore residue [19].

In conclusion, comprehensive and systematic analysis of a number of key residues is necessary to establish the correlation between pore architecture and tuning of the substrate selectivity in grapevine NIPs. Our study goes in this direction and provides some initial insights in the determinants of NIPs functionality. Moreover, the effect of ar/R filter substitution on metalloids selectivity of each grapevine NIP was found to be unique, which may open the question whether the channel has an inherent capacity to transport these substrates or has functionally evolved with physiological requirements. Further studies in planta are required to elucidate the physiological relevance of different NIP isoforms in the context of the whole plant.

## Figures and Tables

**Table 1:** Activation energy (*Ea*) for water and glycerol transport of native VvTnNIP1;1 and VvTnNIP6;1, and their ar/R filter mutant variants.

Strains	Activation Energy for Water Transport (kcal mol^−1^)	Activation Energy for Glycerol Transport (kcal mol^−1^)
pUG35	14.05 ± 0.01	24.30 ± 1.20
VvTnNIP1;1	9.80 ± 0.15	6.93 ± 0.22
NIP1;1W86T	11.68 ± 0.06	11.06 ± 0.69
NIP1;1V206I	11.46 ± 0.02	10.52 ± 0.85
NIP1;1W86T/V206I	11.37 ± 0.03	14.37 ± 0.23
VvTnNIP6;1	11.30 ± 0.4	8.60 ± 0.50
NIP6;1T118W	13.56 ± 0.04	10.93 ± 0.28
NIP6;1I239V	14.09 ± 0.02	11.95 ± 0.06
NIP6;1T118W/I239V	14.38 ± 0.02	13.96 ± 0.35

**Table 2:** List of residues in ar/R selectivity filter and their mutants of grapevine NIPs.

NIPs Homologs	Groups	H2 Helix		H5 Helix		Double Mutants
		ar/R Residues	Mutants	ar/R Residues	Mutants	
VvTnNIP1;1	I	W	W86T	V	V206I	W86T/V206I
VvTnNIP6;1	II	T	T118W	I	I239V	T118W/I239V
VvTnNIP2;1	III	G	G87A	S	S206I	G87A/S206I
VvTnNIP5;1	II	A	A111G	I	I230S	A111G/I230S

## Supplementary Materials

Following Supplementary Materials can be found at https://www.mdpi.com/1422-0067/21/18/6697/s1. Figure S1: Expression and localization of GFP-tagged native grapevine NIPs and their ar/R mutants in H2, H5, H2/H5 helices in the membrane of aqy-null *S. cerevisiae*; Table S1: Primers used in the PCR for Site-directed Mutagenesis in ar/R selectivity filter of grapevine NIPs.
