# Supplementary material for: Insights into the Selectivity Mechanisms of Grapevine NIP Aquaporins
Source: Int J Mol Sci. 2020 Sep 13;21(18):6697. doi: 10.3390/ijms21186697 (PMC7576499; doi:10.3390/ijms21186697)
Supplement: Supplementary file 1 [file ijms-21-06697-s001.pdf]

International Journal of *Molecular Sciences*

MDPI

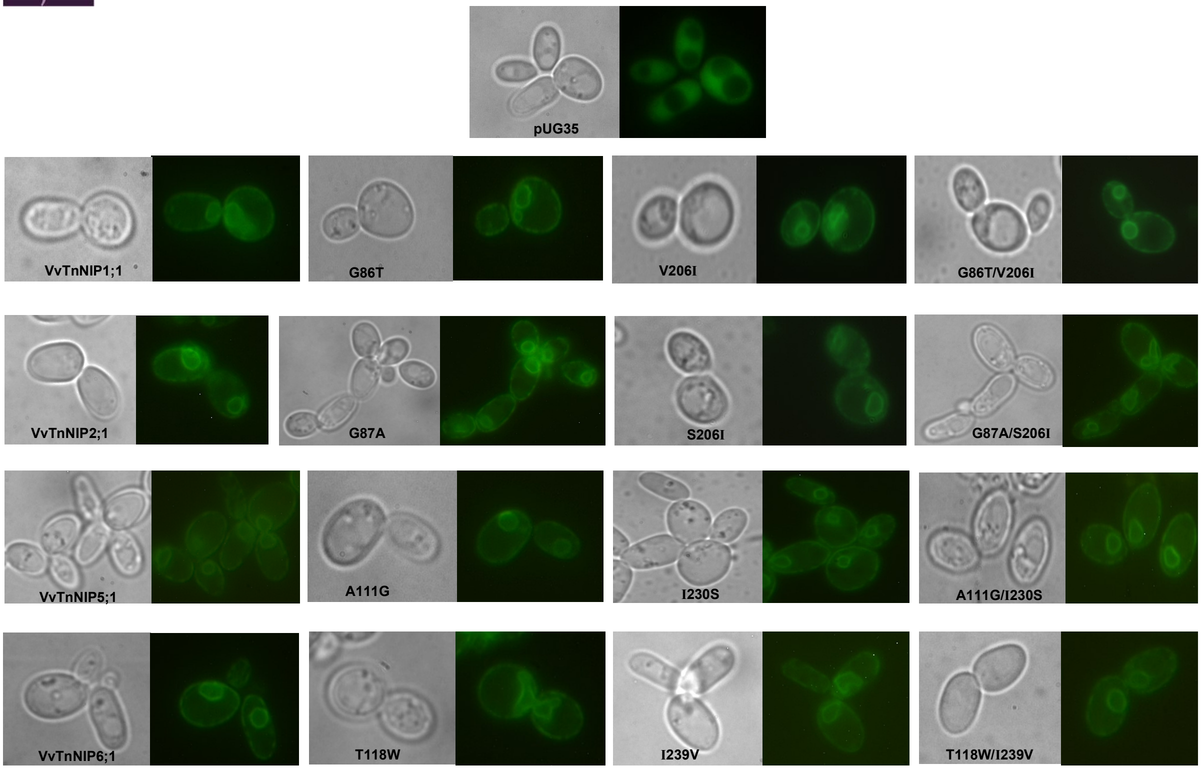

Figure S1. Expression and localization of GFP-tagged native grapevine NIPs and their ar/R mutants in H2, H5, H2/H5 helices in the membrane of aqy-null S. cerevisiae. Left panel show images under phase contrast, whereas right panel pictures are taken under fluorescence microscopy at 100X magnification. Cytosolic accumulation of GFP was observed in yeast cells, transformed with empty plasmid pUG35.

Table S1. Primers used in the PCR for Site-directed Mutagenesis in ar/R selectivity filter of grapevine NIPs.

Primer name	Sequence
NIP1;1 W86T Fwd	GGAATATCCATAGTGACGGGACTGGTTGTGATG
NIP1;1 W86T Rev	CATCACAACCAGTCCCGTCACTATGGATATTCC
NIP1;1 V206I Fwd	GCTACTGTGTTACTTAATATCATGTTTGCAGGGCCA
NIP1;1 V206I Rev	TGGCCCTGCAAACATGATATTAAGTAACACAGTAGC
NIP2;1 G87A Fwd	AAGCTTGGAGCATCGGTTGCAGCTGGACTGATAGTCACAGCTATG
NIP2;1 G87A Rev	CATAGCTGTGACTATCAGTCCAGCTGCAACCGATGCTCCAAGCTT
NIP2;1 S206I Fwd	TCAGCAGTATGCATAACTATCATCTTGGCAGGACCAGTATCAGGT
NIP2;1 S206I Rev	ACCTGATACTGGTCCTGCCAAGATGATAGTTATGCATACTGCTGA
NIP5;1 A111G Fwd	ATAGGGAATGCGGCATGCGGCGGGCTTGCAGTGATGATAGTGATT
NIP5;1 A111G Rev	AATCACTATCATCACTGCAAGCCCGCCGCATGCCGCATTCCCTAT
NIP5;1 I230S Fwd	ACTGTTATGCTGAACAGTCTTGTGGCAGGGCCGTCG
NIP5;1 I230S Rev	CGACGGCCCTGCCACAAGACTGTTCAGCATAACAGT
NIP6;1 T118W Fwd	TCCTGGGGCTGGCAGCCTCCTGGGGGCTGGCGGTGATGGTCATAATAC
NIP6;1 T118W Rev	GTATTATGACCATCACCGCCAGCCCCCAGGAGGCTGCCAGCCCCAGGA
NIP6;1 I239V Fwd	ACTGTCATGCTCAATGTTCTCATCGCCGGGGAAACC
NIP6;1 I239V Rev	GGTTTCCCCGGCGATGAGAACATTGAGCATGACAGT

*Underlined base pairs are for single amino acid substitution.
